# P-148. Melioidosis: A Seven-Year Review (2017-2023)

**DOI:** 10.1093/ofid/ofae631.353

**Published:** 2025-01-29

**Authors:** Deepak Thangaraju, Varun Sundaramoorthy, Vigna Thiagarajan

**Affiliations:** kovai medical center and hospital limited, coimbatore, Tamil Nadu, India; kovai medical center and hospital limited, coimbatore, Tamil Nadu, India; kovai medical center and hospital limited, coimbatore, Tamil Nadu, India

## Abstract

**Background:**

Melioidosis, caused by Burkholderia pseudomallei (B pseudomallei), is a severe disease endemic to Southeast Asia and northern Australia. A high index of clinical suspicion is required for diagnosis. In resource-limited settings, a lack of training and expertise leads to the misidentification of this pathogen. Delay in laboratory diagnosis and non-compliance with treatment can have devastating consequences. A study on the epidemiology of melioidosis will help in training, raising awareness, and improving the disease management. Therefore, this study was done to describe the clinical findings of melioidosis diagnosed at our center in south India.

colonies of Burkholderia pseudomallei on sheep blood agar and MacConkey agar

**Figure:**
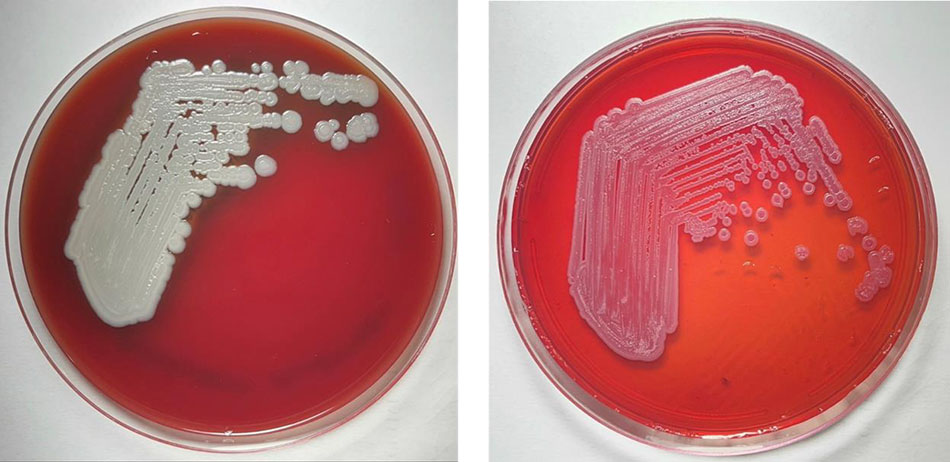

**Methods:**

A retrospective study was done from 2017 to 2023 (7 years). Patients’ data were obtained from medical records. Quantitative variables were expressed as means, and qualitative variable as frequencies.

**Results:**

During the study period, 70 patients were diagnosed with melioidosis. The mean age group was 52 years. Ninety-one percent of them were men. Except for two patients, none of the other patients (97%) were residents of coastal areas. The risk factors were diabetes mellitus (the most common), chronic kidney disease, steroid use, cirrhosis, alcoholism, and others. In 5 patients, more than one organ system was involved. In 43% of patients, the organism was isolated in more than one sample. B pseudomallei was isolated in 71% of patients from the blood, 39% of patients from the musculoskeletal system, 21% of patients from the respiratory system, 9% of patients from the liver and spleen each, 4% of patients from the central nervous system, 3% of patients from ascitic fluid, and urine in one patient (1%). All the isolates were sensitive to ceftazidime. Twenty-three percent of patients died due to the infection.

**Conclusion:**

Our institute is one of the high-volume centers for melioidosis. Diabetes mellitus is the most common risk factor. In contrast to data from other endemic countries, musculoskeletal involvement is the most common in our study. This is probably because of a delay in contacting health care or because the diagnosis is missed during the acute phase of illness, when the pulmonary system is commonly affected. B pseudomallei should be actively looked for while investigating infections in diabetic patients from India.

**Disclosures:**

**All Authors**: No reported disclosures

